# Notice of Dysentery, as It Occurred in Some Parts of Central Ohio

**Published:** 1864-02

**Authors:** Andrew J. Scott

**Affiliations:** Loudonville, Ohio


BUFFALO

Medical and Surgical Journal.

VOL. III. FEBRUARY, 1864. No. 7.

ART. I.—*Notice of Dysentery, as it occurred in some parts of Central Ohio, By* ANDREW J. SCOTT, M. D., *Loudonville, Ohio.*

During the early part of the past summer, we had more than an ordinary number of cases of intermittent and remittent fever, all presenting much of biliary derangement, and frequently accompanied by an extremely irritable condition of the stomach and bowels. These continued to prevail till about the first of July, and then gradually gave way, being soon replaced by an occasional case of *dysentery*, which disease by the first of August had become a regular epidemic. At first prevailing very generally in this and a few neighboring towns, selecting for its victims those of delicate health, particularly children and old people, gradually extending into the surrounding country, growing more malignant in character, attacking in turn and indiscriminately all ages, committing its ravages alike on those living in opulence and in the humble sphere in life, till about the middle of September, having reached its culminating point it began to be more sporadic in character and milder in form, disappearing in the localities where it first appeared, till at last the approach of winter for the present and we fain would hope permanently exempt us from so unwelcome a visitor; one that will long be remembered on account of the vacancies it has caused in many a home circle.

Following as it did malarious disease, periodic in character, it was but natural to infer that it also partook of that character, deriving its origin and existence from the same source, and amenable to the same remedies; but a careful observation of the disease in all its different phases convinced me that it had no connection whatever with malarious poison or malarious disease, unless we attribute all disease to that indescribable something entitled malaria. Instead of being benefited by remedies calculated to arrest ordinary malarious disease, it was aggravated thereby. The symptoms were, as I suppose is the case in all such epidemics, various in character, presenting all the range of peculiarities that we see in the constitutions of patients, and characterized by extreme thirst, a red tongue, nausea, pain in the abdomen, straining at stool, tenesmus and frequent discharges of blood and mucus; but was not accompanied by that amount of fever and general indications of inflammatory action that is spoken of by authors, being fairly entitled to be called *typhoid* in its character, so much so indeed that to meet with a case requiring depleting measures was a rare exception from the ordinary cases; an exception indeed, that did not fall under my personal observation, though I heard of a few falling under the observation of neighboring practitioners. The various modes of treatment as laid down by Watson, Wood and other eminent physicians, were in turn resorted to in my cases, except bleeding, an omission which I have now no reason to regret, and should feel fully as well satisfied had the next powerful depletant, namely, emetics, been left out in the same category, as in the few instances in which I employed them, they did not appear to be of any benefit, and decidedly prostrated my patients, sacrificing the strength, which in the future of the disease they so much needed.

One of my neighbors informed me he cured all his patients by administering at the outset a full dose, say ʒvi. bitart. potass; another that he cured all his, by administering opium and tannin in combination, opii gr. i, tannin gr. iii, every four to six hours; but it was not my fortune to be so successful, though I used these remedies, as well as almost every other mentioned by our standard authors for the treatment of this disease.

The indications, as I conceived in this epidemic, were, to relieve the pain and suffering of the patient, promote the flow of bile, of which there was nearly always not only a deficiency, but an entire absence, arrest the intestinal disease and support the strength. For this purpose I found that opium in some form, calomel, castor oil and brandy, together with cataplasms, warm fomentations, enema, mucilage, and a milk diet, were the best means in my hands; and nothing outside of these remedies appeared to possess much real value, except sub. nit. bismuth, which I have not noticed as a standard remedy in this disease, but which proved exceedingly useful in many cases, allaying irritability of the stomach, and acting as a sedative, affording relief not given by any other medicine. The time required for a given case varied, as might be expected, in proportion to the constitution of the patient and the severity of the attack, ranging from a few days to as many weeks; but generally yielding within ten days to the proper remedies, or about that time assuming grave features and rapidly running its course, to be terminated in death. Many of the cases were mild, and evidently nature possessed the power to throw off the disease; in such cases but little treatment was necessary further than to regulate the diet and enjoin quiet on the patient; this rendered the table of mortality more creditable to the physician. But many of the cases were of the most grave character, the following of which selected from over a hundred which came under my personal observation, will serve to give the reader some idea of the character of this epidemic.

August 27th, was called to see M. R., an intelligent little girl of five years, and learned she had been suffering from a severe attack of dysentery for the last four days, and was then having frequent discharges of bloody mucus, with severe pain, a feeble circulation, thirst and much nervous irritability. She had been under treatment. Ordered opii gr. ss, with calomel gr. i, every four hours, alternated with enema, composed of mutton suet water and five drops of laudanum, mustard cataplasms and warm fomentations.

August 28th. Patient much as when last seen; pulse 140 per minute; had not taken any nourishment; continued treatment, increasing the quantity of laudanum, the anodyne being well borne; and ordered a full dose of castor oil and turpentine at bed time; mutton tea and milk punch to be given to support the strength.

August 29th. The oil had produced free catharsis, changing for a short time the character of the stools, though scarcely ameliorating the unpleasant symptoms, and but little nourishment had been taken. Ordered opii and calomel gr. ss each, every four hours, with anodyne and astringent enema, warm bath and nourishment as before.

August 30th. No change to note, only patient growing weaker; found it had been necessary to increase the quantity of anodyne in enema; that being the only means of securing any rest for patient. Withdrew the mercurial and continued other treatment, urging the free use of milk punch.

August 31st. Patient evidently sinking, extremities cool, pain extreme, and only relieved by anodyne enema; stools more natural; appearance of bile, but little blood, though very frequent, as often as four in an hour. Ordered anodynes as before, occasional large enema of warm water and milk to relieve the tormina and enema containing laudanum when necessary to secure rest; milk punch and other suitable nourishment.

September 1st. Patient sunk down and died early in the day.

In this case there was but little febrile excitement, the surface being rather cool, and the capillary circulation deficient. From the first time I saw this case it gradually failed; each succeeding visit finding it worse no medicine really proving of any service except *anodynes*, which in a measure relieved the distress they could not cure.

*Case 2d*—August 27th. Called to see Dr. ——, aged 64, (who had been the physician first called to the former case which resulted so unhappily in my hands.) He was suffering from an attack of dysentery which had caused him some distress for a day or two, but had attacked him with alarming violence the previous night. This case was one of more than ordinary severity, and in addition to the symptoms common in such cases, was accompanied by the most irritable condition of the stomach imaginable. Ordered calomel gr. xx, to be followed by a full dose of castor oil, mustard cataplasms, warm fomentations, etc.

August 28th. Patient much as when last seen, though the medicine had operated freely as an emetic and cathartic, carrying off a large quantity of bilious matter. Pulse 100 per minute and quite feeble; other symptoms as when last seen. All food had been rejected almost immediately on taking. Ordered opium gr. iii, alternated with sub nit. bismuth gr. iv, and allowed gum water and toast water for drink; warm fomentations continued.

August 29th. No change to note. Continued treatment.

August 30th. Much as when last seen. Continued treatment.

August 31st. No change. Continued treatment, with the addition of gr. ii calomel, every six hours.

September 1st. Ordered a full dose of castor oil and applied a large blister over the stomach and bowels, to be followed by former treatment.

September 2d. Stomach less irritable; other symptoms much as when last noted. Continued the opium and bismuth, and ordered all the milk punch the stomach would tolerate.

September 3d, 4th, 5th, 6th and 7th. No change to note from the 2d. Continued the treatment, moving the bowels in the mean time with castor oil.

September 8th. Discharges less frequent, tongue slightly moist, had rested better during the night; stomach had retained more food. Continued opium and bismuth with an increased quantity of stimulant and nourishment in the shape of milk punch, hot sling, toast, etc.

September 9th. Patient improving; discharges growing less frequent, with but little blood; pulse feeble, requiring stimulant freely. Ordered sufficient anodyne to secure rest.

September 10th. Patient still improving slowly, and went on to a favorable convalescence, being able to resume his professional labors in a measure by the 25th of the month.

*Case 3d*—September 15th. Visited Mrs. F——, aged 60 years. Pulse feeble, and only 50 in a minute; surface cold, tongue dry; stomach irritable, discharges frequent and abundant, consisting of coffee-ground colored substance, mingled with blood; pain and nausea. Ordered calomel gr. ii, opium gr. i, every five hours, with subnit. bismuth gr. iii, at equal distance, with mustard cataplasms, warm fomentations and stimulants.

September 16th. But little change. Continued treatment, diminishing the quantity of opium to gr. one-fourth, the pain being less; and ordered astringent enema.

September 17th. No change worthy of note. Ordered castor oil.

September 18th. The oil had for a while changed the appearance of the stools, but they had again assumed the former character, occurring once in two hours; patient free from pain, though evidently prostrating; pulse only 48 per minute. Ordered acetate lead gr. ii, every four hours with astringent enema and stimulants,

September 29th. Patient evidently sinking; stools as frequent as before; added kino, and continued other treatment, endeavoring to arouse the capillary circulation by friction with dry flannel and washing with nitro-muriatic acid.

September 20th. Much as when last seen, Continued treatment,

September 21st. Patient died.

In this case it may be proper to remark that this attack had supervened upon a former one about three weeks previous, from which she had recovered readily. After the first day she suffered but little pain or thirst; stomach tolerated medicine, ordinarily well; pulse never raised above 50 in a minute. Neither mercurials nor astringents had any effect. Opium produced stupor, consequently was but sparingly administered, stimulants alone appearing of any benefit, and by their free use unquestionably prolonging life.

*Case 4th*—Sept. 4th. Was called to see J. D——, a healthy boy of 7 years, who had the previous night an attack, and was then presenting all the symptoms of a well marked case of dysentery. Ordered calomel gr. viii, to be followed with costor oil.

Sept. 5th. Patient much improved, stools nearly natural. Ordered Dover's powder gr. iii, every six hours.

Sept. 6th. Dismissed patient, supposing him well.

Sept. 9th. Was called again to see him, he being reported, having a relapse; found discharges frequent, with all the dysenteric symptoms manifested in the most aggravated form. Ordered calomel gr. viii, to be followed by castor oil.

Sept. 10th. Medicine had operated, but produced no change. Ordered calomel gr. ss, opium gr. ss, every four hours, alternated with xv. drops of a combination of Tr. digitalis and spirits nitre, and warm fomentations.

Sept. 10th, 11th and 12th. No change to note. Continued treatment, with the occasional addition of a few drops of laudanum.

Sept. 13th. Ordered a full dose of castor oil, to be followed after operating by former treatment.

Sept. 14th, 15th, 16th and 17th. But little change. Continued treatment.

Sept. 18th. Ordered castor oil, to be followed by ten drops of laudanum every five hours.

Sept. 19th, 20th and 21st. Used the laudanum, only giving small quantities of port wine.

Sept. 22d. Ordered calomel and oil, to be followed by laudanum with a small quantity of port wine.

Sept. 23d. Stools bilious, slightly streaked with blood, less frequent, the patient having rested for several hours the past night. Gave only laudanum and wine.

Sept. 24th, 25th and 26th. Patient improving slowly. Continued laudanum and wine with milk diet.

From this time on there were no dysenteric discharges, though he still had some fever and a very irritable condition of the bowels, which persisted until about the 5th of October, which was eventually controlled by the internal administration of nit. silver.

In conclusion it may be proper to add that the disease was contagous in character, though not affecting all who came in contact with it; seldom did it enter a family without making more or less impression on most, if not all its members. While it is a disease that in most cases will yield kindly to treatment, in many cases it appears from the beginning to be of a singularly grave character, bidding absolute defiance to all efforts to even alleviate suffering, on account perhaps of the malignancy of the attack, or some peculiar condition of the system, owing to which, medicines are, either not absorbed at all, or are perverted from their natural influence. Consequently we not unfrequently saw astringents fail to act as such, mercurials to have their specific, or even any effect; while opium and the various anodyne preparations nauseated, and failed to relieve pain, even aggravating an already irritated alimentary canal, producing as it were in an aggravated form the very trouble they were given to relieve. The cases where the disease was confined to the colon and rectum were almost uniformly amenable to treatment, and attended with early and favorable results. It was only where the disease was associated with an irritable condition of the entire alimentary canal, and consequent derangement of the digestive organs, that the disease assumed its gravest type. I am more than ever convinced of the utter contempt that we should feel toward all specifics that are lauded by irregular practitioners in the treatment of this disease, such as *charcoal, leptandrin, etc.;* they may be of use in some cases, and at certain times, but he who lauds them as specifics forfeits his right to an honorable place in the profession.

The writer has not presumed in this article to instruct any member of the profession, but has reported these cases, remembering with what anxiety he perused every late journal that fell under his observation, hoping to find something *new*, that would prove as a beacon-light to a shipwrecked mariner. Therefore this has been written, hoping it may call forth the efforts of some who are able to brace up the younger members of the profession when a fearful epidemic threatens alike to sweep away both patients and reputation.

